# Influence of periodontal ligament heights on sequential and simultaneous maxillary molar distalization using clear aligners

**DOI:** 10.12669/pjms.41.8.11943

**Published:** 2025-08

**Authors:** Rongxiu Zhang, Lin Zhang, Zeyu Wang, Zhigang Wu

**Affiliations:** 1 Rongxiu Zhang Department of Stomatology, The First Affiliated Hospital of Bengbu Medical University, Bengbu, Anhui,233004, P.R. China; 2 Lin Zhang Department of Stomatology, The First Affiliated Hospital of Bengbu Medical University, Bengbu, Anhui,233004, P.R. China; 3 Zeyu Wang Department of Stomatology, The First Affiliated Hospital of Bengbu Medical University, Bengbu, Anhui,233004, P.R. China; 4 Zhigang Wu Department of Stomatology, The First Affiliated Hospital of Bengbu Medical University, Bengbu, Anhui,233004, P.R. China

**Keywords:** Clear aligners, Distalization modes, Finite element analysis, Molar distalization, Periodontal ligament height

## Abstract

**Objective:**

This study investigates the influence of varying periodontal ligament (PDL) heights on maxillary molar distalization using clear aligners (CAs). The analysis emphasizes the biomechanical impacts of PDL height reduction on stress distribution, tooth displacement, and anchorage control across two distalization modes: sequential and simultaneous.

**Methods:**

This study is a retrospective single-case finite element analysis conducted at the First Affiliated Hospital of Bengbu Medical College. Data was obtained from a 26 years old male patient with Angle Class II malocclusion who presented at the hospital on March 19, 2024. Data collection was completed on the same day, and finite element modeling and biomechanical analysis were performed from March 20, 2024 to December 29, 2024. A finite element (FE) model was developed to evaluate molar movement in both sequential mode (where the second molar moved 3.00 mm prior to the first molar’s 0.25 mm movement) and simultaneous mode (where both molars moved 0.25 mm concurrently). Simulations were conducted under three PDL conditions: full height (1.0—control group), moderate reduction (0.67), and severe reduction (0.33). Key biomechanical responses, including stress distribution within the PDL and initial displacement of molars and anterior teeth along three axes (X, Y, Z), were analyzed.

**Results:**

Reduced PDL height significantly amplified stress concentration near the cervical margin of molars and anterior teeth, with uneven stress patterns becoming pronounced under severe PDL reduction (0.33). This led to increased molar tipping and heightened anterior anchorage loss in both modes. Although sequential distalization minimized these adverse effects due to more controlled stress distribution, reduced PDL height remained a critical factor driving greater inefficiency and mechanical instability.

**Conclusions:**

PDL height variations fundamentally influence molar distalization mechanics and anchorage stability, overshadowing the effects of distalization modes. Reduced PDL height intensifies stress imbalances and risks periodontal damage, underscoring the importance of personalized force management strategies in patients with compromised periodontal conditions.

## INTRODUCTION

Maxillary molar distalization, a widely used approach for addressing mild to moderate crowding and Class II malocclusion, is experiencing increased interest with the adoption of clear aligners (CAs). CAs are favored for their aesthetic, comfort, and periodontal health advantages over traditional fixed appliances. For patients with compromised periodontal conditions, such as periodontitis, CAs also reduce inflammation and control subgingival pathogens, making them particularly valuable in clinical management.^1^

However, CAs have limitations in precisely guiding certain tooth movements, particularly in controlling root alignment or managing anteroposterior and vertical corrections.^2^ These biomechanical challenges emphasize the need for improved aligner designs to minimize adverse effects, such as tipping or mesial movement. Finite element analysis (FEA), a widely used computational technique,^3^ plays a crucial role in simulating the mechanical behavior of orthodontic tooth movement, particularly in conditions affected by specific periodontal factors.

The periodontal ligament (PDL) is a connective tissue structure with a thickness varying from 0.15 mm to 0.38 mm, functioning as the primary mediator of force absorption, stress distribution, and tooth stabilization during orthodontic treatment.^4^ Under normal conditions, the PDL balances forces between the root and the alveolar bone, preserving the biomechanical environment required for controlled tooth movement.^5^ However, in cases of reduced PDL dimensions,^4^ the ability of the ligament to evenly redistribute stress diminishes, leading to concentrated forces and increased risks for tipping or anchorage loss, presenting challenges in treatment planning.

Existing research on maxillary molar distalization using CAs primarily explores factors such as anchorage designs,^6^,^7^attachment configuration,^8^ degrees of distalization,^9^ and materials engineering properties,^10^ while often neglecting the impact of PDL conditions and variations in PDL height. By combining FEA modeling and molar distalization protocols, this study addresses the gap by investigating how biomechanical performance changes with different PDL dimensions. The goal is to elucidate how varying PDL heights influence stress distribution, anchorage stability, and tooth displacement dynamics.

This research was specifically designed to provide actionable insights into mechanically adaptive solutions for patients with reduced PDL support, enhancing precision and safety for those managing conditions such as periodontitis or long-term bone degradation.

## METHODS

This study is a retrospective single-case finite element analysis, with data obtained from a 26 years old male patient with Angle Class II malocclusion who presented at the Department of Stomatology, First Affiliated Hospital of Bengbu Medical College on March 19, 2024. Informed consent was obtained from the patient. Data collection was completed on the same day as the patient’s visit, and finite element modeling and biomechanical analysis were performed in the hospital’s Biomedical Engineering Laboratory from March 20, 2024 to December 29, 2024. The patient exhibited permanent dentition, a complete dental arch, deep overbite, deep overjet, and extracted third molars. Using Mimics 21.0 and Geomagic Wrap 2021, an initial STL model of the maxilla and teeth was refined into a STEP format for analysis. To simulate structural details, a PDL width of 0.25 mm was applied by offsetting the tooth surfaces, alongside a clear aligner model with a 0.75 mm film thickness.

### Ethical Approval:

The research conformed to the guidance of the World Medical Association Helsinki Declaration for biomedical research involving human subjects, and the imaging data involved was approved by the institutional review boards (protocol number: [2025]KY004, date: January 16, 2025).

### Inclusion & Exclusion Criteria:

In this study, the inclusion criteria consisted of healthy maxillary molar models without prior orthodontic treatment or periodontal disease, while the exclusion criteria included models with significant anatomical anomalies or prior dental treatments that could affect the results.

### Submodel generation:

The PDL model with normal height was used as the baseline, and submodels with reduced PDL heights (67% and 33% of original height) were created in SolidWorks by removing corresponding solid volumes.^11^

### Material properties and Meshing:

Elastic modulus and Poisson’s ratio were assigned as per literature,^12^ modeling the materials as homogeneous, isotropic, and linearly elastic. Differences between cortical and cancellous bone, along with enamel and dentin, were disregarded for simplification. Using Ansys Workbench, tetrahedral meshing was applied with mesh sizes of 2.0 mm (maxilla), 1.0 mm (teeth and aligners), and 0.15 mm (PDL), resulting in 299,717 nodes and 158,715 elements.

### Boundary and Contact conditions:

The maxillary top surface was fixed to ensure stability, while other conditions included bonded interfaces between the tooth root, PDL, and alveolar bone, and no-separation contact between adjacent teeth. A friction coefficient of 0.2 was set for interactions between the aligner and crown surfaces. These constraints were designed to replicate realistic force transmission without sliding.

### Mechanical loading:

Static forces from the clear aligner were simulated based on initial misalignments and corresponding aligner designs.^13^ Distal movement protocols were examined via two schemes: sequential distalization (second molar moved 3.00 mm followed by the first molar by 0.25 mm) and simultaneous distalization (both molars moved 0.25 mm together). Six submodels were constructed, differentiated by PDL height and movement schemes.^7^,^14^ In this study, we employed two modeling scenarios: sequential modes (models A1, B1, and C1) and simultaneous modes (models A2, B2, and C2). The analysis is conducted under three conditions: full height (1.0—control group), moderate reduction (0.67, including models B1 and B2), and severe reduction (0.33, including models C1 and C2).

### Coordinate system and Observation indicators:

A local 3D coordinate system was used for precise measurements of crown and root movements. Reference points included incisal/cusp tips and root apices for incisors, canines, molars, and premolars. Analysis focused on the right maxilla due to symmetry. Data recorded included initial displacements (X, Y, Z) and equivalent stress distribution within the PDL across submodels.

## RESULTS

### PDL hydrostatic stress:

**Fig.1:**
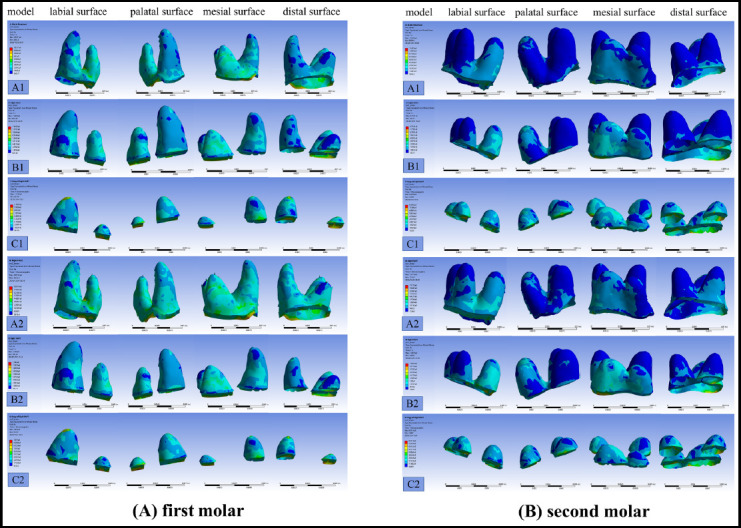
The stress distribution in the periodontal ligament of maxillary molars.

Stress distributions in the PDL of maxillary molars varied markedly with changes in PDL height. In the full-height PDL model (1.0 condition), stress was more uniformly distributed and primarily concentrated near the cervical margin and furcation areas of the mesiobuccal and palatal roots. **Fig.1** However, as PDL height was reduced (0.67 and 0.33 conditions), localized peak stress increased significantly, shifting toward the cervical margin and amplifying uneven stress distribution. Simultaneous distalization resulted in broader and higher stress regions compared to the sequential approach, while both modes exhibited similarly deteriorated stress uniformity with decreasing PDL height.

This figure evaluates the movement of maxillary molars in sequential and simultaneous modes. Stress levels are represented with a color scale, where red areas denote maximum stress concentrations and blue areas indicate minimum stress regions. The left side depicts the first molar in Figure A, while the right side shows the second molar in Figure B, with stress distribution illustrated for the mesial, distal, buccal, and palatal surfaces.

**Fig.2:**
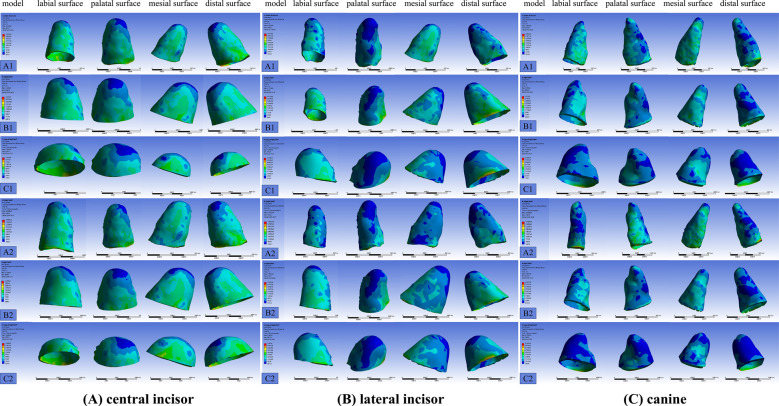
The stress distribution in the periodontal ligament of maxillary anterior teeth.

In maxillary anterior teeth, localized stress increased with declining PDL height. **Fig.2** For the full-height model, stress was concentrated around the cervical palatal margins of central and lateral incisors and the palatal surfaces of canines. Reduced PDL height (0.67 and 0.33) led to greater stress imbalances, particularly along cervical labial regions. Canines experienced consistently higher stress compared to incisors, confirming their critical role as anchorage support teeth. Simultaneous distalization yielded higher stress concentrations than sequential mode, with marked effects under severe PDL reduction.

This figure illustrates the movement of maxillary anterior teeth (central incisor in Figure A, lateral incisor in Figure B, and canine in Figure C) under the previously mentioned modeling scenarios. The color scale indicates stress levels, with regions of high stress shown in red and low stress in blue. Stress distribution is presented for the facial, lingual, and proximal surfaces.

**Fig.3:**
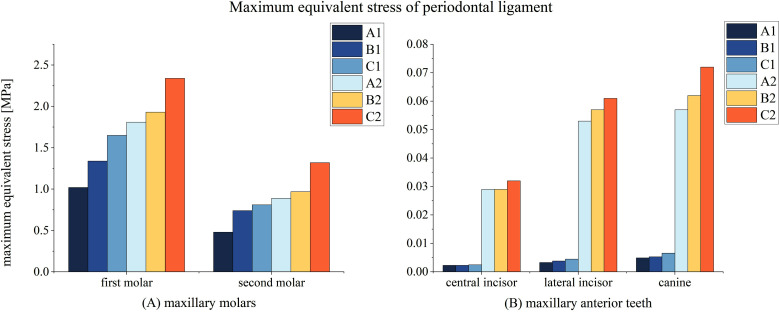
Maximum Equivalent Stress in the Periodontal Ligament.

Across all models, peak stress increased significantly with decreasing PDL height, consistent for both molars and anterior teeth.**Fig.3** In the molar region, the first molar exhibited the highest stress, particularly in the 0.33 PDL height group, where cervical overload risks became notable. Similarly, anterior teeth, particularly canines, bore the brunt of anchorage stress under reduced PDL height. Although simultaneous distalization produced higher stress levels, the overall trend of greater peak stress with decreasing PDL height was observed in both modes.

This figure presents the maximum equivalent stress for molars in Panel A and anterior teeth in Panel B in the sequential and simultaneous modes described earlier. Each tooth type’s stress distribution is mapped to show how loading sequences and periodontal ligament height reduction impact stress responses.

**Fig.4:**
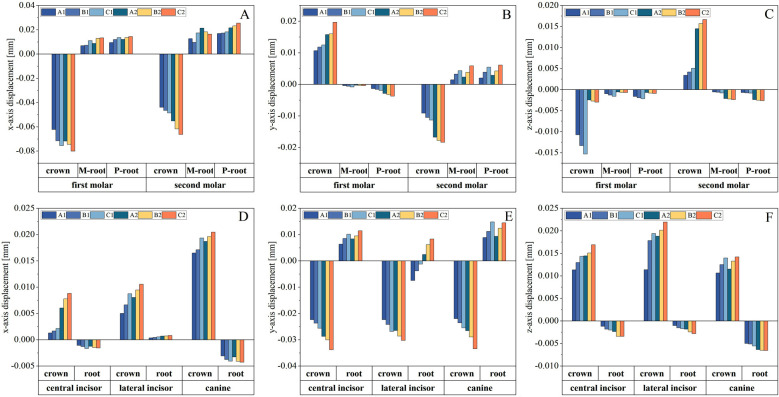
Initial Displacement of Maxillary Anterior Teeth in Three Dimensions.

Molars displayed distinct displacement patterns in all three-dimensional axes, with variations correlated with PDL height and distalization mode. Fig.4A, Fig.4B, C. Along the mesiodistal direction (X-axis), molars exhibited distal crown tipping across all PDL conditions. This tipping effect was magnified as the PDL height decreased, with the simultaneous distalization mode producing higher distalization efficiency but also more pronounced crown mesial tipping compared to the sequential method. Buccolingual movement (Y-axis) showed greater irregularities under reduced PDL height. For the first molar, lower PDL height exacerbated crown buccal tipping and root lingual movement, while the second molar displayed opposing directional tipping, with crown mesiolingual tipping and root buccal displacement, indicating weakened control of buccolingual motion in both movement modes. Vertical displacement (Z-axis) revealed contrasting patterns, with crown extrusion in the first molar and significant intrusion in the second molar. These vertical effects were amplified as PDL height decreased, particularly in the 0.33 condition. Simultaneous distalization consistently produced greater crown extrusion and intrusion compared to sequential movement, further accentuating imbalances under compromised periodontal conditions.

This figure illustrates the three-dimensional initial displacement of maxillary molars (A for X-axis, B for Y-axis, and C for Z-axis) and anterior teeth (D for X-axis, E for Y-axis, and F for Z-axis), indicating the effects of various displacement modes and periodontal ligament area on tooth movement. Positive X-axis values indicate mesial movement, while negative values indicate distal movement. Positive Y-axis values represent palatal movement, and negative values indicate buccal movement. Positive Z-axis values indicate occlusal movement, while negative values indicate apical movement.

Maxillary anterior teeth demonstrated mesial crown tipping and labial movement as anchorage. Fig.4D, Fig.4E, F. These tendencies increased under reduced PDL levels (0.67 and 0.33), with lateral incisors and canines being most affected. Under simultaneous distalization, labial tipping was significantly greater than sequential movement, and crown intrusion became more pronounced, especially in the severe PDL reduction group (0.33 condition). Anchorage control became increasingly compromised under these conditions, suggesting that reduced PDL height heightened tooth displacement instability.

## DISCUSSION

Through static finite element analysis, this study quantitatively reveals for the first time the impact mechanism of periodontal ligament (PDL) height changes on the biomechanics of maxillary molar distalization using clear aligners (CAs).^15^ When PDL height decreases to 0.33, the unevenness of stress distribution significantly increases (concentrated around the neck of the teeth)^12^, leading to a heightened risk of molar tipping.^16^ This finding aligns with Geramy’s (2002) theory of PDL stress concentration.^17^ Although previous research (such as that by Gao et al.^8^) has focused on optimizing attachment design for treatment efficiency, their models did not account for the dynamic effects of PDL height. This study clarifies the core position of periodontal biomechanics in orthodontic treatment by quantifying the relationship between PDL height and stress distribution, and further emphasizes the necessity of extending static finite element models to four dimensional dynamic analysis (4D FEA) to simulate the time-varying processes of PDL remodeling and bone adaptation during long-term treatment, thus addressing the oversimplified assumptions of biological response associated with current models.

In clinical practice, these findings support the development of tiered treatment strategies: for patients with healthy PDL structures, simultaneous distalization can shorten treatment duration under the premise of enhanced anchorage (e.g., using mini-implants)^18^-^20^ and real-time monitoring, but caution must be taken regarding the risk of periodontal damage caused by stress concentration. For patients with reduced PDL height (especially with significant alveolar bone loss), sequential distalization improves the uniformity of stress distribution through phased application of force, decreasing the likelihood of molar tipping. Furthermore, for patients with compromised PDL, optimizing aligner design (such as using gingivally-extended rectangular attachments to disperse neck stress or customizing aligner shapes via topological optimization algorithms)6-78 can further enhance treatment safety. The proposed strategies provide a practical framework for orthodontic decision-making under complex periodontal conditions.

### Limitations

Firstly, the static finite element model did not integrate the dynamic remodeling of PDL and cellular mechanical response, potentially underestimating the biological complexity during long-term treatment. Secondly, the simulation results need to be further validated through prospective clinical studies (e.g., comparing periodontal indicators between sequential and simultaneous distalization groups). Lastly, there is a need to develop a 4D FEA framework that integrates temporal dimensions and multi-scale biological responses^21^, such as incorporating the mechanosensing mechanisms of periodontal membrane fibroblasts to predict bone remodeling rates and risk thresholds under different PDL conditions.

## CONCLUSION

In summary, PDL height, as a key biomechanical parameter, directly influences the efficacy and safety of clear aligners. This study provides a theoretical basis for its clinical management through quantitative analysis and promotes the evolution of orthodontic treatment from a “one-size-fits-all” model toward precision and personalization^22^

### Authors Contribution:

***RZ and LZ:* Study design, literature search, manuscript writing**.

***ZW and ZW:* Collected the data, performed the analysis and did Review**.

All authors have read and approved the final manuscript and are responsible for the integrity of the study.
